# Adeno-associated virus-mediated inhibition of ROCK2 promotes synaptogenesis and neurogenesis in rats after ischemic stroke

**DOI:** 10.4103/NRR.NRR-D-24-01474

**Published:** 2025-06-19

**Authors:** Liuliu Shi, Ting Zhu, Chengyan Ge, Yongkun Yang, Qi Wan, Shifang Li

**Affiliations:** 1 Department of Neurosurgery, The Affiliated Hospital of Qingdao University, Qingdao, Shandong Province, China; 2 Institute of Neuroregeneration & Neurorehabilitation, School of Basic Medicine, Qingdao Medical College, Qingdao University, Qingdao, Shandong Province, China; 3 Faculty of Life and Health Sciences, Shenzhen University of Advanced Technology, Shenzhen, Guangdong Province, China

**Keywords:** adeno-associated virus, axonal regeneration, gene therapy, ischemic stroke, neurogenesis, neurological recovery, neuronal survival, neuroplasticity, Rho-associated kinase 2, synaptogenesis

## Abstract

Neurite outgrowth and synaptogenesis are critical steps for functional recovery following ischemic stroke. Damaged axons of the central nervous system in adult mammals exhibit limited regenerative capacity, resulting in enduring neurological deficits. Recent findings from our research indicate that inhibition of Rho-associated kinase (ROCK)2 facilitates neuroprotection in different models of central nervous system diseases. In addition, our prior studies have demonstrated that axonal protection enhances the regeneration of injured axons. However, it remains unclear whether the axonal protection mediated by ROCK2 inhibition also facilitates synaptogenesis. In this study, we aimed to investigate the effects of inhibiting ROCK2 expression on synaptogenesis and neurogenesis in ischemic stroke using an shRNA-expressing adeno-associated virus (AAV) vector (AAV-sh.ROCK2). We demonstrated that AAV-sh.ROCK2 increased neurite outgrowth and facilitated synaptogenesis *in vivo*. Furthermore, AAV-sh.ROCK2 increased neuronal survival and promoted neurogenesis following middle cerebral artery occlusion surgery as well as long-term motor functional recovery after ischemia/reperfusion injury. Notably, AAV-sh.ROCK2 also stimulated serotonergic and dopaminergic axon sprouting after ischemia/reperfusion injury. Mechanistically, AAV-sh.ROCK2 activity resulted in increased anti-collapsin response mediator protein 2 activation and reductions in RhoA and ROCK2 expression. Our study identified ROCK2 as a critical regulator of synaptogenesis and neurogenesis, highlighting it as a promising target to facilitate neuroprotection and regeneration in ischemic stroke.

## Graphical abstract

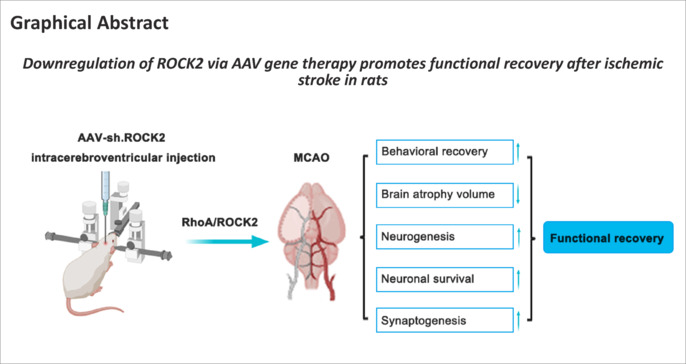

## Introduction

Stroke encompasses both ischemic and hemorrhagic types and continues to be one of the leading causes of death and disability in adults globally (Feigin et al., 2022; Hilkens et al., 2024; Xie et al., 2024b). The incidence of stroke is escalating yearly, largely because of the growing aging population (Koh and Park, 2017; Katan and Luft, 2018), with ischemic stroke accounting for 87% of all cases (Tsao et al., 2022; Xie et al., 2024a). Currently, the primary therapeutic interventions for ischemic stroke include recombinant tissue plasminogen activator and endovascular thrombectomy (Hilkens et al., 2024). However, owing to the narrow treatment time window and potential hemorrhagic transformation (Kim, 2019), fewer than 10% of patients derive benefit from these treatments. Although hundreds of neuroprotective approaches have been developed and evaluated, few can be used clinically because of unsatisfactory treatment outcomes (Nemoto, 2023), which leads to high disability and mortality rates of ischemic stroke and places an enormous burden on families and society (Zhou et al., 2019; Wang et al., 2024b; Xie et al., 2024b). Hence, new treatment strategies to decrease mortality and reverse neurological deficits after ischemic stroke are urgently needed.

Neurological recovery after ischemic stroke is significantly limited in adult mammals, primarily because of the death of neurons and the release of axonal inhibitory molecules from damaged nerve myelin, leading to neural network damage. Neural network recovery depends on neuroplasticity, which consists of neurogenesis and synaptogenesis. Neurogenesis and synaptogenesis in the infarction area are the key reasons for the reconstruction of neural networks. The main areas involved in neurogenesis in the adult brain are the subventricular zone (SVZ) of the lateral ventricle and the subgranular zone of the hippocampal dentate gyrus (Qin et al., 2022). Our previous research revealed that neural stem cells (NSCs) and neural precursor cells (NPCs) in the SVZ can deviate from the conventional rostral migratory stream and migrate to the ipsilateral cortex and striatum after ischemic stroke (Donega and Raineteau, 2017). These cells can regenerate axons, form synapses, differentiate into mature neuronal phenotypes, and rebuild neural networks. These processes are crucial to realize neural function remodeling after ischemic stroke. However, only a small percentage of these cells survive for prolonged periods (Thored et al., 2006). Therefore, exploring the mechanism by which neuroplasticity is promoted is highly important for the treatment and prognosis of ischemic stroke patients.

Synaptogenesis is primarily induced by the release of substantial quantities of inhibitory molecules, such as myelin-associated glycoproteins, chondroitin sulfate proteoglycans, oligodendrocyte myelin glycoprotein, and neurite growth inhibitor-A, from damaged nerve myelin (Schwab and Strittmatter, 2014; Zhang et al., 2020). These structurally distinct proteins all bind to the neurite growth inhibitor receptor, which inhibits axonal outgrowth by activating RhoA and its target Rho-associated kinase (ROCK) (Fujita and Yamashita, 2014; Zhang et al., 2020; Wang et al., 2022b). Inhibition of the RhoA/ROCK signaling pathway improves axonal regeneration and neuronal survival (Koch et al., 2014). Thus, the RhoA/ROCK signaling pathway is a central factor in inhibiting axonal regeneration in adult mammals.

ROCK belongs to the serine-threonine kinase family and plays key roles in the regulation of neuronal injury and survival, axonal guidance, and neuron regeneration (Gao et al., 2022). ROCK1 and ROCK2 are different ROCK isomers. ROCK1 is expressed mainly in nonneuronal tissues, while ROCK2 is clearly expressed in the brain and spinal cord, and its expression increases with age (Martín-Cámara et al., 2021). Up-regulation of the ROCK2 subtype is thought to be a RhoA/ROCK pathway activation marker in the brain (Lu et al., 2020). ROCK2 inhibitors have a positive effect on neurite regeneration, damaged neuron survival, and hippocampal neurogenesis (Tönges et al., 2012; Christie et al., 2013; Li and Liu, 2019; Xu et al., 2022). However, these drugs are not completely target-specific, and blood-brain barrier permeability is poor, resulting in hypotension, intracranial hemorrhage, and abnormal hepatic and renal function (Mueller et al., 2005; Koch et al., 2018). Therefore, they cannot be effectively applied in clinical practice. Previous studies have shown that down-regulation of ROCK2 expression with shRNA-expressing adeno-associated virus (AAV) vectors for the treatment of optic nerve injury, spinal cord damage, and Parkinson’s disease can promote nerve function recovery, axon growth, synaptic formation, and survival of injured neurons (Koch et al., 2014; Challagundla et al., 2015; Saal et al., 2015).

Therefore, we used RNA interference methods and an shRNA-expressing AAV9 vector (AAV-sh.ROCK2) to evaluate the influences of ROCK2 on synaptogenesis, neurogenesis, and long-term motor functional recovery in rats after ischemic stroke.

## Methods

### Animals and experimental protocol

Forty male Sprague-Dawley rats (8 weeks old, weight 220–250 g, and specific-pathogen-free grade) were purchased from Pengyue Animal Breeding Co., Ltd. (Jinan, China, license No. SCXK (Lu) 2022-0006). Estrogen has been shown to have a neuroprotective effect on ischemic stroke (Wang et al., 2022a). Therefore, to exclude the influence of estrogen and more accurately study the specific mechanism of cerebral ischemia, researchers use male rats for middle cerebral artery occlusion (MCAO) models. In addition, the vascular anatomy of male rats is relatively clear, which helps to improve the success rate of surgery and the reliability of the model. The rats were housed in individual cages with controlled temperature (22 ± 2°C) and humidity (55% ± 5%) conditions and provided with sufficient food and water under a 12-hour light/dark photocycle. All rats were adapted to their new environment for 3 days before the experiment. All animal experimental procedures were carried out following the guidelines of the Animal Ethics Committee of Qingdao University (approval No. 20230710SD4020241115129, approval date: June 19, 2023) and were conducted in strict accordance with the National Institutes of Health Guide for the Care and Use of Laboratory Animals (8^th^ ed., National Research Council, 2011). All experiments were designed and reported according to the Animal Research: Reporting of *In Vivo* Experiments (ARRIVE) guidelines (Percie du Sert et al., 2020).

**Figure 1:**
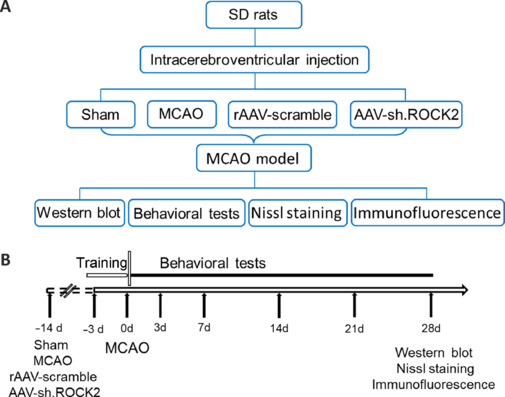
Schematic diagram of the experimental design protocols. AAV: Adeno-associated virus; MCAO: middle cerebral artery occlusion; SD: Sprague-Dawley.

The experimental protocol is shown in **Figure 1**. Forty rats were randomly divided into four groups: the sham group (no MCAO, *n* = 10), MCAO group (MCAO, *n* = 10), rAAV-scramble group (MCAO + rAAV-scramble, *n* = 10), and AAV-sh.ROCK2 group (MCAO + AAV-sh.ROCK2, *n* = 10). Rats in the sham group were subjected to operations similar to those in the experimental groups, but they were not given intraventricular injections, and sutures were not introduced into the carotid artery. The AAV-sh.ROCK2 group and rAAV-scramble group were injected with AAV-sh.ROCK2 and rAAV-scramble 14 days before being subjected to MCAO. The MCAO group, rAAV-scramble group, and AAV-sh.ROCK2 group were subjected to MCAO surgery for 90 minutes, followed by suture removal to establish cerebral blood flow reperfusion to the ischemic brain tissue. The rats were anesthetized with isoflurane (RWD Life Science, Shandong, China) by inhalation during the surgery. The isoflurane concentrations were 4% and 2% for induction and maintenance of anesthesia, respectively. Anesthesia was considered successful when the rat did not respond to a tail pinch. The rats were euthanized after anesthesia with pentobarbital sodium (100 mg/kg in phosphate-buffered saline, PBS, GLPBIO, Montclair, CA, USA), and brain tissues were collected for western blot (WB) analysis, Nissl staining, and immunofluorescence staining 28 days after surgery.

### Cloning and adeno-associated virus vector production

All AAVs were synthesized by Shanghai Jikai Gene Company, Shanghai, China. Neuron-specific expression was driven by the hSyn1 promoter, and the ROCK2-expressing shRNA gene was inserted into an expression plasmid carrying a neuron-specific hSyn1 promoter by molecular cloning, which was designated pSUPER-hSyn-ROCK2-shRNA. A scrambled shRNA without any target gene was used as a negative control for AAV-sh.ROCK2. The sequences of the shRNA primers used were as follows: ROCK2-shRNA forward primer: 5′-GAT CCC CTG CAA AGT TTA TTA TGA TAT ACT TCC TGT CAT ATA TCA TAA TAA ACT TTG CAT TTT TGG AAA-3′. ROCK2-shRNA reverse primer: 5′-AGC TTT TCC AAA AAT GCA AAG TTT ATT ATG ATA TAT GAC AGG AAG TAT ATC ATA ATA AAC TTT GCA GGG-3′.

### Intracerebroventricular injection

After anesthesia, scissors were used to remove the hair from the rats’ heads, after which they were placed on a stereotaxic instrument, followed by disinfection of the skin in the surgical area. The skull was exposed through a midline surgical incision, and the fontanel was located, with the anterior fontanel used as the zero point of stereotactic positioning. Stereotaxic coordinates were used to locate the right lateral ventricle (1.0 mm behind the bregma, 1.5 mm right lateral to the midline, and 4.0 mm deep in the skull) (Do et al., 2024). Afterward, a craniotomy was performed at the stereotaxic coordinates. A total volume of 10 μL of PBS containing 2 × 10^10^ AAV-sh.ROCK2 or rAAV-scramble viral particles was injected into the right lateral cerebral ventricle at a rate of 0.4 μL/minute through a 10 µL Hamilton syringe. The needle was held in place for 10 minutes to avoid backflow after the injection was finished. Finally, the bone hole was blocked with bone wax, and the incision was sutured and disinfected after needle extraction.

### Construction of a focal cerebral ischemia/reperfusion injury model

The MCAO model was generated as described previously (Zhu et al., 2024; Wang et al., 2025). Briefly, an incision was made in the middle of the skin on the neck after the rats were anesthetized. Then, the neck muscles were separated to reveal the right common carotid artery, external carotid artery (ECA), and internal carotid artery, and a suture (L3600, Jialing, Guangzhou, China) was inserted 22 mm from the right ECA into the right internal carotid artery until it occluded the origin of the middle cerebral artery. Ninety minutes after occlusion, the suture was withdrawn to establish reperfusion, and the incision was closed after ligation of the ECA. After MCAO surgery, tramadol (2.5 mg/kg) was injected into the tail vein to reduce postoperative pain in the rats. The same operation was performed in the sham group, except for suture insertion. The success of the MCAO model was measured by laser Doppler flowmetry (PeriCan PSIZ, Sweden). The standard for a successful MCAO model was that the cerebral blood flow after the operation was reduced by 70% compared with that before the operation. During the surgery, the temperature was maintained at 37 ± 0.5°C with a temperature-controlled heating pad. Neurological deficits after MCAO were evaluated using the Longa method as follows: 0, no neurological deficits observed; 1, the rat was unable to fully extend the left forelimb; 2, the rat circled to the left while walking; 3, the rat fell to the left side while walking; 4, the rat could not walk automatically or exhibited loss of consciousness. Rats scoring between 1 and 3 points were selected for later experiments (Hu et al., 2024).

### Behavioral tests

The rats were trained 3 days before the MCAO model was established, and the baseline was set 1 day before MCAO model establishment. A modified neurological severity score (mNSS) evaluation, rotarod test, and grip test were performed on the 3^rd^, 7^th^, 14^th^, 21^st^, and 28^th^ days after MCAO. All behavioral tests evaluated motor and sensory functions and were performed by a researcher who was blinded to the experimental design and treatment.

#### Modified neurological severity score

As previously described (Wang et al., 2024a), the mNSS includes motor, sensory, reflex, and balance tests to assess neural function in rats. The scores range from 0 to 18, and a higher score indicates more serious neurological injury (0, no deficit; 1–6, mild deficit; 7–12, moderate deficit; 13–18, severe deficit).

#### Rotarod test

The rotarod test was carried out to evaluate balance and coordination of the rats (Jia et al., 2023). Briefly, the rats were placed on a rotarod device (HUAYON, Shenzhen, China) and allowed to run. The rotational speed was accelerated from 5 rotations/minute to 40 rotations/minute within 5 minutes. The time when the rats fell off the rotating rod was recorded, with a cutoff time of 5 minutes. Rats were trained on the rotating rod 3 days before MCAO surgery, and it was ensured that every rat learned the procedure. The rats were tested three times on the 3^rd^, 7^th^, 14^th^, 21^st^, and 28^th^ days after MCAO, with an interval of 15 minutes between each test, and the average latency to fall was recorded.

#### Grip strength test

To assess forelimb motor dysfunction, the left forelimb grip strength of the rats was tested using a grip strength meter with a modified previously reported method (Ren et al., 2024). The left forelimb of the rat was placed on the grip strength meter (HUAYON). When the rat grabbed the bar with the left forelimb, the rat’s tail was pulled back until the grip was broken. The test was performed three times, and the average grip strength was recorded to estimate the left forelimb grip strength. The rats were tested on the 3^rd^, 7^th^, 14^th^, 21^st^, and 28^th^ days after MCAO surgery.

### Weight percentage

The rats were weighed 1 day before surgery and measured on the 3^rd^, 7^th^, 14^th^, 21^st^, and 28^th^ days after being subjected to MCAO. The weight percentage was calculated as post-operative body weight/pre-operative body weight.

### Western blotting

The rats were euthanized and transcardially infused with 100 mL PBS 28 days after being subjected to MCAO. Their brains were quickly removed and frozen in a –80°C freezer. The ischemic cortex and striatum (including the ischemic area) were cleaved in radioimmunoprecipitation assay buffer containing a protein phosphatase inhibitor, a phosphatase inhibitor, and phenylmethanesulfonyl fluoride for 30 minutes at 4°C to completely cleave the protein. The tissue lysate was centrifuged at 12,000 × *g* for 15 minutes at 4°C and the supernatant was collected. The protein content of the supernatant was determined using a BCA kit (GLPBIO). The supernatant was subsequently adjusted to a uniform concentration of 4 μg/μL and boiled at 100°C for 10 minutes. Proteins (20 μg) were loaded onto a sodium dodecyl-sulfate polyacrylamide gel electrophoresis gel (Meilunbio, Liaoning, China) (10%–12%) for electrophoresis and subsequently transferred onto polyvinylidene fluoride membranes (Millipore, Billerica, MA, USA). Then, the membranes were incubated in blocking buffer (5% skim milk, Phygene, Fuzhou, China) for 1 hour at room temperature, incubated at 4°C overnight with rabbit anti-ROCK2 (1:1000, HUABIO, Zhejiang, China, Cat# ER1706-48, RRID: AB_3069063), rabbit anti-collapsin response mediator protein 2 (CRMP2) (1:1000, Proteintech, Wuhan, China, Cat# 14684-1-AP, RRID: AB_10858228), rabbit anti-Ras homolog A (RhoA) (1:1000, Immunoway, Suzhou, China, Cat# YM8050), rabbit anti-growth-associated protein 43 (GAP43) (1:1000, Abcam, Cambridge, UK, Cat# Ab-75810, RRID: AB_1310252), β-actin (1:10,000, HUABIO, Cat# EM21002, RRID: AB_2819164), and glyceraldehyde 3-phosphate dehydrogenase (GAPDH) (1:8000, HUABIO, Cat# R1210-1, RRID: AB_3073206), and then incubated with the appropriate secondary antibody, Goat anti-Rabbit IgG antibody (1:10,000, HUABIO, Cat# HA1001, RRID: AB_2819166), at room temperature for 1 hour. Finally, target protein bands on the membrane were detected via enhanced chemiluminescence (Meilunbio), and the data were analyzed using ImageJ software (version 1.53q, NIH, Bethesda, MD, USA); β-actin and GAPDH were used as internal controls.

### Evaluation of the cerebral cortical width index

We used the cortical width index to measure cerebral cortical expansion. The rats were euthanized 28 days after being subjected to MCAO and then transcardially infused with PBS and 4% paraformaldehyde, followed by collection of the brain. Whole-brain photos were taken with a camera (Nikonz30, Nikon, Tokyo, Japan), and the width from the midpoint of the brain to the limbic brain was measured. The right/left cortical width ratio was defined as the cortical width index.

### Nissl staining and measurement of brain atrophy volume

The brains were collected 28 days after the rats were subjected to MCAO, preserved in 4% paraformaldehyde for 1 night, and then submerged in 30% sucrose for 72 hours at 4°C. The brain tissues were cut into 35 μm slices with a freezing microtome. Six brain slices spanning the whole injury area were collected continuously, and Nissl staining (Beyotime, Shanghai, China) solution was used to evaluate the volume of brain atrophy. The brain atrophy volume was calculated by integrating the brain atrophy area in each slice with the distance between the various layers. The atrophy volume (V) of each slice was measured using ImageJ software and calculated with the following formula: V = Σ (Ai × Ts × n), where Ai is the ischemic area measured in the brain slice, Ts is the thickness of the brain slice (35 μm), and n is the number of brain slices between two adjacent levels. The brain atrophy percentage [(contralateral hemispheric volume – ipsilateral non-infarcted volume)/contralateral hemispheric volume] was used for statistical analysis (Lei et al., 2023).

### Brain tissue preparation and immunofluorescence staining

The brains were collected 28 days after the rats were subjected to MCAO, preserved in 4% paraformaldehyde for 1 night, submerged in 30% sucrose for 72 hours at 4°C, snap frozen, and stored at –80°C until sectioning. The brains were cut into 35 μm slices with a freezing microtome (Leica, Wetzlar, Germany). Brain slices within 1 to –1 mm of the bregma were collected and stored in antigen protective solution, which was composed of 50% PB, 25% glycol, and 25% glycerol.

Immunofluorescence staining was used to assess the expression of different neural markers in the brain. The brain sections were washed three times with PBS, permeabilized with 0.3% Triton X-100 for 30 minutes, and blocked with 10% bovine serum albumin for 2 hours at room temperature. The sections were then incubated with rabbit anti-GAP43 (1:500, Abcam, Cat# Ab-75810, RRID: AB_1310252), rabbit anti-synaptophysin (SYP) (1:500, HUABIO, Cat# ET1606-56, RRID: AB_3069749), rabbit anti-doublecortin (DCX) (1:800, Cell Signaling Technology, Danvers, MA, USA, Cat# 4604S), rabbit anti-Nestin (1:800, Cell Signaling Technology, Cat# 89529S), and rabbit anti-neuronal nuclei (NeuN) (1:500, Abcam, Cat# ab-177487, RRID: AB_2532109) primary antibodies at 4°C overnight. All brain sections were subsequently incubated with the corresponding secondary antibody at room temperature for 60 minutes. The secondary antibodies included Alexa Fluor 647-conjugated goat anti-rabbit IgG (1:1000, Cell Signaling Technology, Cat# 4414S) and Alexa Fluor 594-conjugated goat anti-rabbit IgG (1:1000, Bioss, Beijing, China, Cat# bs-0295D-BF594). The cell nuclei were stained with 4′,6-diamidino-2-phenylindole (DAPI) (Solarbio, Beijing, China, Cat# No. C0065). Image information was collected using a confocal microscope and a fluorescence microscope (Nikon). ImageJ software was used to calculate the number of immunopositive cells in the ipsilateral SVZ and striatum region of each rat in the different groups. At least three brain sections from each group were analyzed, and the results are expressed as the percentage of immunopositive cells relative to that in the sham group.

### Statistical analysis

All statistical analyses were performed with GraphPad Prism software version 8.0.1 (GraphPad Software, San Diego, CA, USA, www.graphpad.com). All data are expressed as the mean ± standard deviation. Student’s *t*-test was used to compare two groups of experimental data. Statistical comparisons among multiple groups were performed using one-way analysis of variance followed by the least significant difference test to analyze changes in the cerebral cortical width index, brain atrophy volume, and WB and immunofluorescence staining data. Two-way analysis of variance followed by the least significant difference test were used to analyze the mNSS, rotarod test, grip strength test, and weight percentage among different groups. Three independent trials were performed for each experiment; *P* < 0.05 was considered statistically significant.

## Results

### Successful transfection of adeno-associated virus and effective knockdown of ROCK2 expression in the rat brain

**Figure 2:**
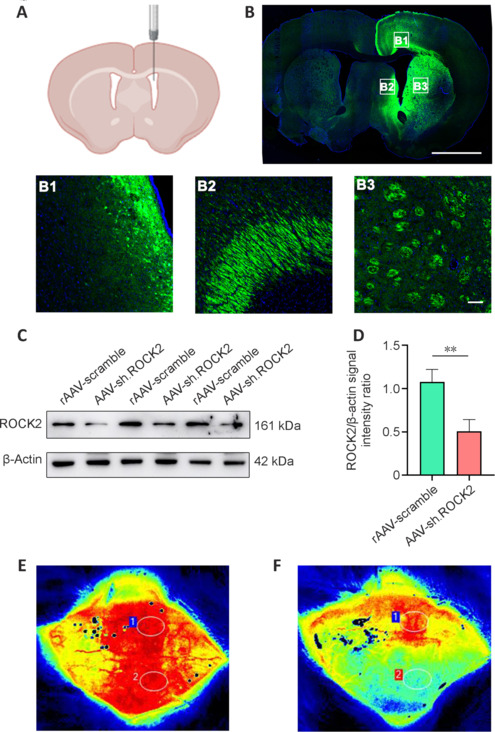
Successful transfection of AAV-sh.ROCK2 by intraventricular injection into the brain tissue of rats subjected to MCAO, effectively knocking down ROCK2 protein expression. (A) Intraventricular injection mode diagram. (B) Brain sections showing adeno-associated virus (AAV)-mediated enhanced green fluorescent protein (EGFP) expression (Alexa Fluor 488, green) after AAV injection into the right lateral cerebral ventricle at 6 weeks. B1, B2, and B3 show AAV-mediated EGFP expression in the cortex, subventricular zone, and striatum. Scale bars: 1000 μm. (C, D) Representative western blot bands (C) and quantification (D) of ROCK2 expression in the ischemic cortex and striatum on the 28^th^ day after MCAO in rats. (E) Cerebral blood flow before MCAO. (F) Cerebral blood flow after MCAO (red indicates abundant blood flow, and blue indicates insufficient blood flow). Data are presented as the mean ± SD, *n* = 3. **P < 0.01 (Student’s *t*-test). AAV: Adeno-associated virus; EGFP: enhanced green fluorescent protein; MCAO: middle cerebral artery occlusion; ROCK2: Rho-associated protein kinase 2.

To induce AAV9 to successfully transfect and effectively knockdown ROCK2 expression in the brain, we constructed the hSyn promoter-EGFP-MIR155 (MCS)-SV40 PolyA. Two weeks before MCAO, AAV-sh.ROCK2 was injected into the right cerebral ventricle (**Figure 2A**). The rats were euthanized 6 weeks after AAV9 injection, and the brains were collected for frozen sectioning. All AAV9 vectors expressed enhanced green fluorescent protein (EGFP), allowing for visual confirmation that the stereotactic injection of the virus successfully transduced the SVZ, cortex, and striatum (**Figure 2B**). To ensure that ROCK2 was effectively knocked down, striatum and cortex tissues were collected for WB analysis. The results revealed that ROCK2 was effectively knocked down at the protein level (**Figure 2C** and **D**). To confirm that the MCAO model was successfully established, cerebral blood flow before and after the MCAO operation was measured via laser Doppler flowmetry. The cerebral blood flow in postoperative rats was obviously decreased compared with that in preoperative rats, which demonstrated that the MCAO model was successfully established (**Figure 2E** and **F**). The above results show successful transfection of AAV-sh.ROCK2 and effective knockdown of ROCK2 expression in the rat brain.

### AAV-sh.ROCK2 treatment improves long-term neurological recovery in rats subjected to middle cerebral artery occlusion

**Figure 3:**
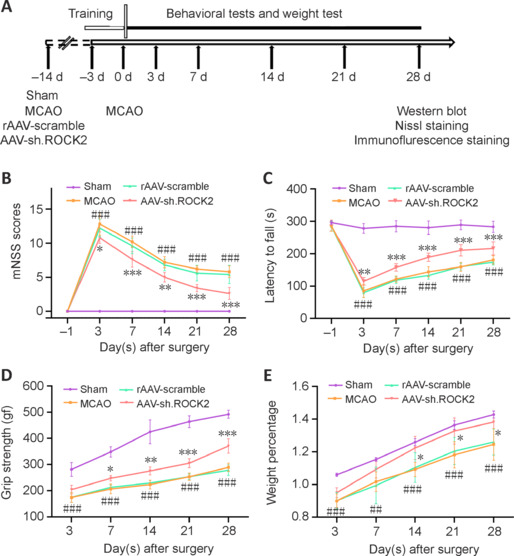
AAV-sh.ROCK2 treatment improves long-term neurological deficits in rats subjected to MCAO. (A) Experimental design diagram. The modified neurological severity score (mNSS) test, rotarod test, grip strength test, and weight percentage were quantified on the 3^rd^, 7^th^, 14^th^, 21^st^, and 28^th^ days after MCAO. (B) mNSS test. (C) Rotarod test. (D) Grip strength test. (E) Weight percentage. Data are presented as the mean ± SD, *n* = 5. ##*P* < 0.01, ###*P* < 0.001, MCAO group *vs.* sham group, *P < 0.05, **P < 0.01, ***P < 0.001, AAV-sh.ROCK2 *vs.* rAAV-scramble (two-way analysis of variance followed by the least significant difference test). AAV: Adeno-associated virus; MCAO: middle cerebral artery occlusion; mNSS: modified neurological severity score; ROCK2: Rho-associated protein kinase 2.

To evaluate the effect of ROCK2 knockdown on the long-term prognosis of neurological function after intraventricular injection of AAV-sh.ROCK2 in MCAO rats, a series of behavioral tests were performed at different time points (**Figure 3A**). With the exception of the sham group, rats in all other groups presented different degrees of neurological deficits, and their neurological function gradually recovered over time after MCAO.

The mNSS is a method to evaluate sensory and motor function in rats. The mNSS values were greater in rats after MCAO surgery than those in the sham group, and the scores improved with increasing postoperative time. These findings indicated that neurological function was damaged after the MCAO operation and that some neurological functions recovered with time. However, the AAV-sh.ROCK2 group presented improved neurological performance, especially long-term performance, at different time points compared with that in the MCAO group and the rAAV-scramble group (*P* < 0.05; **Figure 3B**). The rotarod test was used to measure the balance and coordination of rats. All groups spent a significantly shorter time on the rotarod than did the sham group. The time on the rotarod was gradually prolonged with increasing postoperative time, especially in the AAV-sh.ROCK2 group (*P* < 0.01; **Figure 3C**). The grip strength test was used to assess forelimb motor dysfunction. The grip strength of the left forelimb was obviously decreased compared with that in the sham group, but the grip strength of the AAV-sh.ROCK2 group was obviously increased compared with that of the MCAO group and rAAV-scramble group (*P* < 0.05; **Figure 3D**). As shown in **Figure 3E**, the changes in body weight at different times were recorded. The weight percentages in the sham group increased significantly in a time-dependent manner, while the percentages of body weight in the AAV-sh.ROCK2 group were higher than those in the MCAO group and rAAV-scramble group over time (*P* < 0.05). These results indicated that ROCK2 knockdown after AAV-sh.ROCK2 injection into the ventricle could improve the long-term sensory and motor dysfunction caused by ischemic stroke, which may contribute to reducing the sequelae of permanent motor disability and improving the outcomes of ischemic stroke.

### AAV-sh.ROCK2 treatment reduces brain atrophy volume and increases the cortical width index in rats subjected to middle cerebral artery occlusion

**Figure 4:**
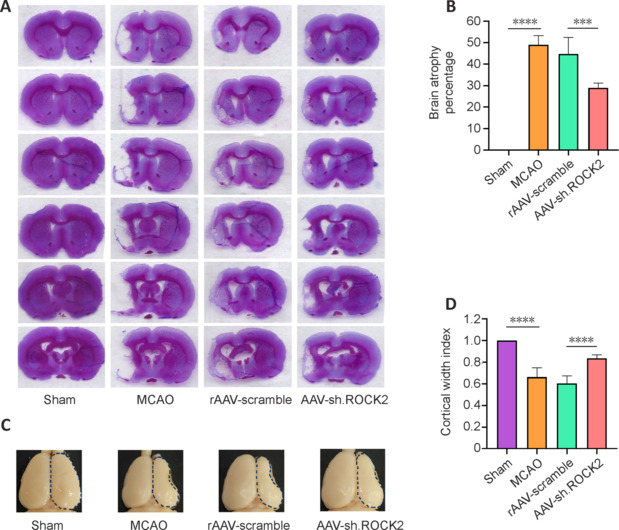
AAV-sh.ROCK2 treatment reduces the percentage of brain atrophy volume and increases the cortical width index in rats subjected to MCAO. (A) Evaluation of brain atrophy in representative photographs of brain slices with Nissl staining. (B) Quantification of the percentage of brain atrophy volume. (C) AAV-sh.ROCK2 treatment increased the cortical width index. The dotted circle represents outline of the ischemic brain tissue. (D) Quantification of the cortical width index. Data are presented as the mean ± SD, *n* = 5. ***P < 0.001, ****P < 0.0001 (one-way analysis of variance followed by the least significant difference test). AAV: Adeno-associated virus; MCAO: middle cerebral artery occlusion; ROCK2: Rho-associated protein kinase.

To investigate the effect of AAV-sh.ROCK2 treatment on the long-term prognostic histology in rats subjected to MCAO, we measured the brain atrophy percentage and the cortical width index 28 days after MCAO surgery. Different degrees of brain atrophy occurred in all groups compared with that in the sham group after MCAO surgery. The brain percentage atrophy was not significantly different between the MCAO group and the rAAV-scramble group. However, AAV-sh.ROCK2 treatment reduced the brain atrophy percentage compared with that in the rAAV-scramble group (*P* < 0.001; **Figure 4A** and **B**). The cortical width index was used to evaluate expansion of the cerebral cortex. AAV-sh.ROCK2 treatment increased the cortical width index compared with that in the rAAV-scramble group (*P* < 0.0001; **Figure 4C** and **D**). These data illustrated that the beneficial effect of the AAV-sh.ROCK2 treatment on ischemic brain tissue outcomes was sustained into the chronic stage.

### AAV-sh.ROCK2 treatment inhibits the RhoA/ROCK2 pathway and promotes collapsin response mediator protein 2 and growth-associated protein 43 expression in rats subjected to middle cerebral artery occlusion

**Figure 5:**
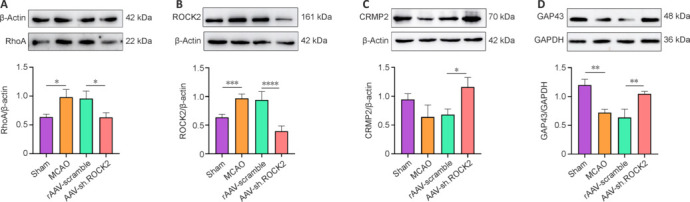
RhoA, ROCK2, CRMP2, and GAP43 protein expression in the ischemic cortex and striatum. Representative western blot bands and quantitative analysis of the protein bands of RhoA (A), ROCK2 (B), CRMP2 (C), and GAP43 (D) in the ischemic cortex and striatum on the 28^th^ day after MCAO in rats, with β-actin used as the reference. Data are presented as the mean ± SD, *n* = 3. *P < 0.05, **P < 0.01, ***P < 0.001, ****P < 0.0001 (one-way analysis of variance followed by the least significant difference test). CRMP2: Collapsin response mediator protein 2; GAP43: growth-associated binding protein 43; RhoA: Ras homolog gene family member A, ROCK2: Rho-associated protein kinase 2.

To study the effect of AAV-sh.ROCK2 treatment on the RhoA/ROCK2 signaling pathway, we examined RhoA and ROCK2 expression at the protein level in rats 28 days after MCAO. The WB results revealed that RhoA and ROCK2 expression increased after MCAO (**Figure 5A** and **B**), indicating that ischemia/reperfusion injury can induce activation of the RhoA/ROCK2 signaling pathway. Compared with the rAAV-scramble group, the AAV-sh.ROCK2 group presented reductions in RhoA (*P* < 0.05; **Figure 5A**) and ROCK2 (*P* < 0.001; **Figure 5B**) expression after MCAO. The RhoA/ROCK2 signaling pathway was inhibited by AAV-sh.ROCK2 treatment. CRMP2 is a downstream substrate of ROCK2 that promotes axonal outgrowth by promoting microscopic assembly. Activation of the ROCK2 signaling pathway leads to CRMP2 phosphorylation and inactivation, which leads to growth cone collapse and inhibition of axonal outgrowth. To determine the effect of AAV-sh.ROCK2 treatment on axonal outgrowth, we evaluated CRMP2 expression at the protein level. The WB results revealed that compared with that in the rAAV-scramble group, CRMP2 protein expression in the AAV-sh.ROCK2 group was obviously increased (*P* < 0.05; **Figure 5C**). The GAP43 is an important molecular marker for synaptic plasticity. To evaluate the effect of AAV-sh.ROCK2 treatment on GAP43 protein expression, the WB results revealed that the GAP43 level was significantly lower in the MCAO group than in the sham group (*P* < 0.01; **Figure 5D**). There was no significant difference between the MCAO group and the rAAV-scramble group. However, the AAV-sh.ROCK2 group presented a significantly greater GAP43 protein expression level than that in the rAAV-scramble group (*P* < 0.01; **Figure 5D**). These results suggested that AAV-sh.ROCK2 treatment may reduce CRMP2 phosphorylation and promotes GAP43 protein expression by inhibiting the RhoA/ROCK2 pathway, thereby further promoting axonal outgrowth and synaptogenesis and improving the long-term prognosis of ischemic stroke patients.

### AAV-sh.ROCK2 treatment promotes neurogenesis in rats subjected to middle cerebral artery occlusion

**Figure 6:**
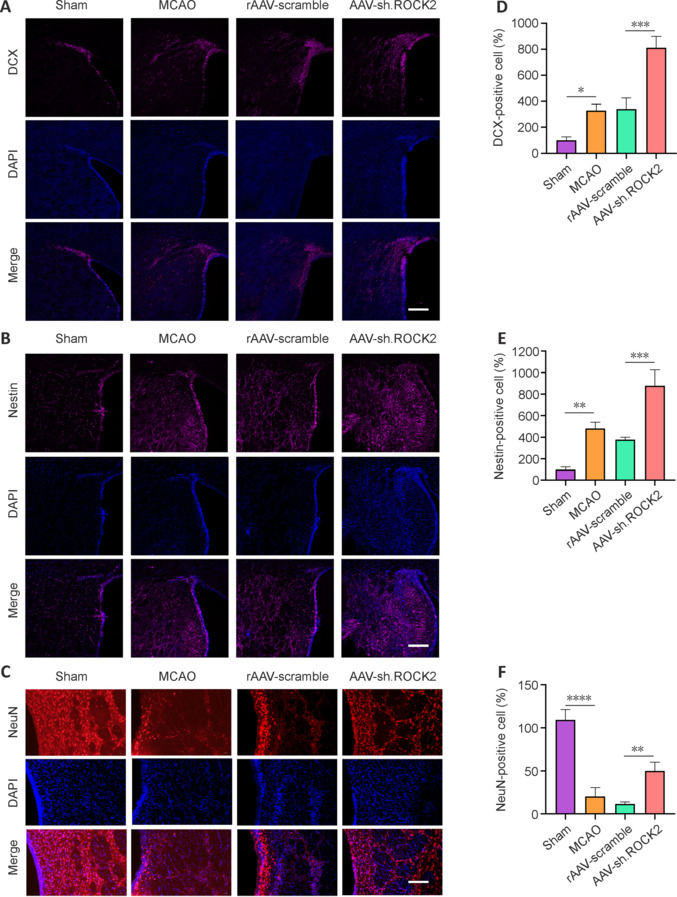
AAV-sh.ROCK2 treatment promotes neurogenesis and neuronal survival in rats subjected to MCAO. (A) Representative confocal immunofluorescence images of doublecortin (DCX)-positive cells (immature neuron marker, Alexa Fluor 647, purple) in the sham, MCAO, rAAV-scramble, and AAV-ROCK2 groups at 4 weeks after ischemic stroke. Cell nuclei were labeled blue with DAPI, and DCX-positive cells were labeled purple using Cy5. Scale bar: 100 μm. (B) Representative confocal immunofluorescence images of Nestin-positive cells (proliferation marker, Alexa Fluor 647, purple) in the sham, MCAO, rAAV-scramble, and AAV-sh.ROCK2 groups at 4 weeks after ischemic stroke. Cell nuclei were labeled blue with DAPI, and Nestin-positive cells were labeled purple using Cy5. Scale bar: 100 μm. (C) Representative confocal immunofluorescence images of NeuN-positive cells (neurons, Alexa Fluor 594, red) in the sham, MCAO, rAAV-scramble, and AAV-sh.ROCK2 groups at 4 weeks after ischemic stroke. Cell nuclei were labeled blue with DAPI, and NeuN-positive cells were labeled red with Cy3. Scale bar: 50 μm. (D) The percentage of DCX-positive cells relative to that in the sham group. (E) The percentage of Nestin-positive cells relative to that in the sham group. (F) The percentage of NeuN-positive cells relative to that in the sham group. Data are presented as the mean ± SD, *n* = 3. *P < 0.05, **P < 0.01, ***P < 0.001, ****P < 0.0001 (one-way analysis of variance followed by the least significant difference test). AAV: Adeno-associated virus; Cy5: Cyanine 5 maleimide; DAPI: 4′,6-diamidino-2-phenylindole; DCX: doublecortin; MCAO: middle cerebral artery occlusion; NeuN: neuronal nuclei; ROCK2: Rho-associated protein kinase 2.

To evaluate the effects of AAV-sh.ROCK2 treatment on neurogenesis, we examined neurogenesis in the ipsilateral SVZ and striatum. The recovery of neurological function after MCAO is limited, mainly because of a decline in neuroplasticity. Neurogenesis is a core factor of neuroplasticity. The SVZ is the main region of neurogenesis in adult mammals, and newly generated NSCs/NPCs can migrate from the SVZ to the ischemic striatum after ischemic stroke, where they differentiate into mature neurons (Liu et al., 2021). Although many NSCs/NPCs are produced in the SVZ and migrate to the ischemic region after ischemic stroke, only a small number of them can differentiate and survive in the long term (Thored et al., 2006). A previous study has shown that ROCK2 inhibition promotes neurogenesis and neuronal survival (Zhai and Guo, 2022). Twenty-eight days after MCAO surgery, we assessed the expression of the neurogenesis markers Nestin (a specific marker of NSCs) and DCX (a marker of immature neurons) in the SVZ and striatum by immunofluorescence staining. MCAO significantly increased DCX-positive cells (*P* < 0.05; **Figure 6A** and **D**) and Nestin-positive cells (*P* < 0.01, **Figure 6B** and **E**) compared with those in the sham group in the ipsilateral SVZ and striatum area. The difference between the MCAO group and the rAAV-scramble group was not statistically significant, but the numbers of DCX-positive cells (*P* < 0.001, **Figure 6A**) and Nestin-positive cells (*P* < 0.001; **Figure 6B**) in the AAV-sh.ROCK2 group were significantly higher than those in the rAAV-scramble group. These data indicate that MCAO stimulated neurogenesis in the SVZ and striatum and that neurogenesis was further increased after AAV-sh.ROCK2 treatment (**Figure 6A** and **B**).

To evaluate the neuroprotective effect of ROCK2 on neurons after ischemic stroke, brain sections from the SVZ and striatum were immunolabeled with a NeuN antibody. Neuronal death is the main cause of impaired nerve function in ischemic stroke. Previous studies have shown that ROCK2 inhibition can promote neuronal survival after spinal cord injury and optic nerve injury (Koch et al., 2014; Challagundla et al., 2015). Compared with those in the sham group, many neurons in the MCAO group died, leading to neural dysfunction. The number of NeuN-positive cells in the SVZ and striatum was significantly greater in the AAV-sh.ROCK2 group than in the rAAV-scramble group (*P* < 0.01). These results suggested that AAV-sh.ROCK2 improved neuronal survival in the ischemic region after ischemic stroke (**Figure 6C** and **F**).

### AAV-sh.ROCK2 treatment promotes synaptogenesis in rats subjected to middle cerebral artery occlusion

**Figure 7:**
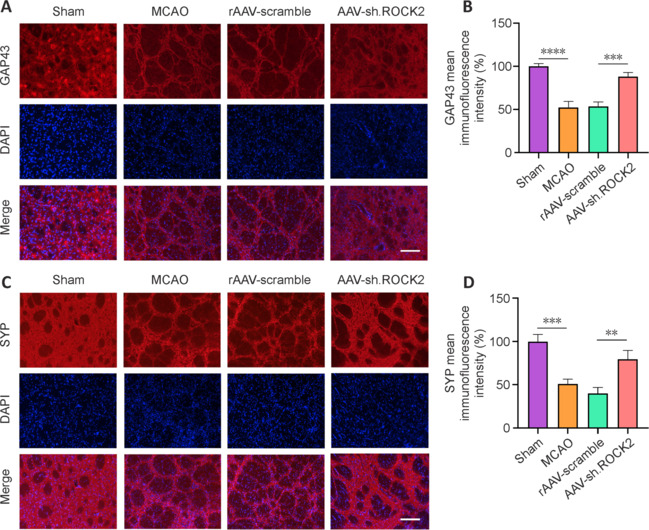
AAV-sh.ROCK2 treatment increases GAP43 and SYP levels after MCAO surgery in rats. (A) Representative confocal immunofluorescence images of GAP43 (synaptic plasticity marker, Alexa Fluor 594, red) in the sham, MCAO, rAAV-scramble, and AAV-ROCK2 groups at 4 weeks after ischemic stroke. Cell nuclei were labeled blue with DAPI, and GAP43 was labeled purple with Cy3. Scale bar: 50 μm. (B) The percentage of GAP43 relative to that in the sham group. (C) Representative confocal immunofluorescence images of SYP (synaptic plasticity marker, Alexa Fluor 594, red) in the sham, MCAO, rAAV-scramble, and AAV-sh.ROCK2 groups at 4 weeks after ischemic stroke. Cell nuclei were labeled blue with DAPI and SYP was labeled purple with Cy3. Scale bar: 50 μm. (D) The percentage of SYP relative to that in the sham group. Data are presented as the mean ± SD, *n* = 3. **P < 0.01, ***P < 0.001, ****P < 0.0001 (one-way analysis of variance followed by the least significant difference test). AAV: Adeno-associated virus; Cy3: Cyanine 3 maleimide; DAPI: 4′,6-diamidino-2-phenylindole; GAP43: growth-associated protein; MCAO: middle cerebral artery occlusion; ROCK2: Rho-associated protein kinase 2; SYP: synaptophysin.

NSCs and NPCs migrating from the SVZ to the ischemic striatum following neurogenesis possess the capacity for synaptogenesis, enabling the formation of synapses and the reconstruction of neural networks. This process is crucial for functional reorganization after ischemic stroke. Therefore, we examined the expression of GAP43 and SYP by immunofluorescence, which are important molecular markers related to synaptic plasticity. We determined the GAP43 immunofluorescence staining intensity in the striatum region. The immunofluorescence results revealed that, compared with that in the sham group, the fluorescence intensity of GAP43 in the MCAO group and rAAV-scramble group was significantly lower (*P* < 0.0001). The fluorescence intensity of GAP43 in the striatum was increased after AAV-sh.ROCK2 treatment (**Figure 7A** and **B**). SYP is a marker used to evaluate synaptic regeneration and remodeling in rats subjected to MCAO. SYP detection by immunofluorescence staining revealed that SYP expression was markedly lower in the MCAO group than in the sham group (*P* < 0.001). These results indicated that synaptic function was impaired after MCAO. There was no significant difference between the MCAO group and the rAAV-scramble group, but the number of SYP-positive cells in the AAV-sh.ROCK2 group was significantly higher than that in the MCAO group or rAAV-scramble group (*P* < 0.01; **Figure 7C** and **D**). Thus, AAV-sh.ROCK2 treatment may play a neuroprotective role by promoting synaptogenesis.

## Discussion

Ischemic stroke remains one of the leading causes of disability and death in adults (Liu et al., 2025). The main cause of disability after cerebral ischemia is an impaired neural network. Therefore, repairing a damaged neural network is the ultimate goal after ischemic stroke. The repair of neural networks in the peri-infarct area is closely related to neurogenesis and synaptogenesis. Therefore, exploring ways to promote neurogenesis and synaptogenesis in the treatment of ischemic stroke is highly important. Myelin-associated neurite growth suppressor proteins limit axonal remodeling and neural function recovery after central nervous system injury (Lu et al., 2018), which is associated with activation of the RhoA/ROCK2 pathway. The RhoA/ROCK2 signaling pathway is closely related to cytoskeletal rearrangement, cell growth, cell migration, proliferation, and development (Fujita and Yamashita, 2014). Therefore, inhibition of ROCK2 expression is considered a potential therapeutic approach to modify the effects of ischemic stroke (Sladojevic et al., 2017).

AAVs are increasingly recognized for their potential in gene therapy because of their low virulence and immunogenicity and their ability to facilitate long-term expression of therapeutic genes *in vivo*. These properties make AAVs ideal carriers for the delivery of exogenous genes, allowing for sustained therapeutic effects without significant adverse immune responses. The safety profile of AAVs has been well documented in clinical trials that have demonstrated their efficacy in treating various genetic disorders (Berns and Srivastava, 2019). Hence, this study investigated the therapeutic effects of AAV-sh.ROCK2 on the long-term prognosis of ischemic stroke.

RhoA/ROCK2 expression increases after ischemic stroke (Zhang et al., 2021; Zhou et al., 2021) and can cause neuronal cell loss, growth cone collapse, and axonal damage. The WB results in this study revealed that the RhoA and ROCK2 expression levels were still high at 28 days after ischemic stroke, which caused the RhoA/ROCK2 signaling pathway to be continuously activated, and the damage caused by ischemic brain injury persisted. We used AAV9, which can induce stable expression *in vivo* for at least 6 weeks, to carry the foreign genes of sh.ROCK2 to specifically knock down ROCK2 expression in the brains of rats subjected to MCAO. RhoA and ROCK2 protein expression was effectively knocked down at 28 days, and the RhoA/ROCK2 signaling pathway was inhibited. Thus, AAV-sh.ROCK2 therapy may be a potential approach to knock down RhoA and ROCK2 expression during chronic ischemic stroke.

Histological and behavioral outcomes are necessary to evaluate the long-term prognosis of ischemic stroke and are the primary parameters for determining whether treatment options can be advanced to clinical trials (Fisher et al., 2009; Fluri et al., 2015; McBride et al., 2015). Therefore, we used the mNSS, rotarod test, and grip strength test to investigate neurological impairment in rats after being subjected to MCAO. The mNSS is one of the most commonly used scores to assess neural function after MCAO modeling in rats and can reflect the neural function deficits of rats from different aspects such as sensory, motor, reflex, and balance tests (Wang et al., 2024a). The rotarod test and grip strength test indicate brain function impairment from different perspectives (Jia et al., 2023; Ren et al., 2024). In our study, the motor function of the rats significantly deteriorated after MCAO, indicating that brain function was significantly impaired. Treatment with AAV-sh.ROCK2 improved neurological impairment at different time points after cerebral ischemia, especially in the chronic stage. Cortical neurological injury induces motor dysfunction in MCAO rats, resulting in quantitative and qualitative impairments in obtaining food (Zhou et al., 2021). We found that the weight percentages of rats subjected to MCAO were significantly lower than those of the sham group, suggesting that the cerebral cortex was damaged, which led to neurological deficits and weight loss. However, the weight of rats subjected to MCAO increased significantly after AAV-sh.ROCK2 treatment.

The death of a large number of neurons is the main factor in nerve function injury after ischemic stroke. The brain tissue atrophies, and cortical cavitation occurs with prolonged time. Previous studies have shown that ROCK2 inhibition can protect neuronal survival after central nervous system injury (Koch et al., 2014; Challagundla et al., 2015). NeuN immunofluorescence staining revealed that ROCK2 promoted the survival of neurons in the ischemic peripheral region. Studies have shown that ROCK2 inhibitors can reduce the cerebral infarction volume (Lee et al., 2014; Li and Liu, 2019) because of the difficulty of classifying infarcts histologically in a long-term MCAO model. Therefore, we calculated the percentage of brain atrophy volume and the cortical width index, which revealed that AAV-sh.ROCK2 treatment reduced both. These data suggest that ROCK2 can protect against ischemic stroke by promoting neuronal survival and reducing brain tissue defects.

The recovery of neurological function after ischemic stroke is associated with various factors, including neuroprotection (e.g., neuronal survival and neuroinflammation) and neuroplasticity (e.g., neurogenesis and protrusion). Neurogenesis is essential for the recovery of nerve function, and ischemic stroke triggers neurogenesis in the SVZ, which generates NSCs/NPCs that migrate to the ischemic striatum and cerebral cortex, which is the basis for the recovery and remodeling of brain function (Sun et al., 2020a). Although a large number of new NSCs/NPCs are generated and migrate from the SVZ to the striatum after cerebral ischemia, only a small number of them differentiate and survive in the long term (Thored et al., 2006; Huttner et al., 2014). Therefore, enhancing endogenous neurogenesis and promoting the survival of newly generated neurons are important for the recovery of neural function after ischemic stroke. Inhibition of the RhoA/ROCK pathway can induce neurogenesis in the adult hippocampus, increase the survival rate of newly generated neurons, and contribute to the recovery of brain function after brain injury (Sandelius et al., 2018; Willis et al., 2024). ROCK inhibitors have also been shown to trigger neurogenesis and increase the survival of new neurons in Parkinson’s disease models (Tönges et al., 2012; Li and Liu, 2019). Therefore, we investigated the effects of AAV-sh.ROCK2 treatment on neurogenesis after ischemic stroke in this study. Nestin and DCX are widely used as markers in different studies to assess neurogenesis (Asgari Taei et al., 2021; Zheng et al., 2024). Immunofluorescence staining results revealed that MCAO led to the proliferation of Nestin-labeled NSCs and DCX-labeled NPCs in the SVZ and their migration to the ischemic striatum. However, only a few NSCs/NPCs survived for up to 4 weeks, but AAV-sh.ROCK2 treatment significantly increased the number of NSCs/NPCs, demonstrating that AAV-sh.ROCK2 treatment could promote neurogenesis and NSC/NPC survival.

Neurogenesis and synaptogenesis are important components of neuroplasticity. Synaptogenesis occurs when newly generated neurons migrate from the SVZ to the ischemic striatum after neurogenesis and reconstruct the neural network, which plays an important role in functional reconstruction after ischemic stroke. To evaluate the effect of AAV-sh.ROCK2 treatment on synaptogenesis after ischemic stroke, we evaluated the expression of GAP43 and SYP, which are important molecular markers related to neuroplasticity. They play important roles in axonal growth and synapse formation during neuronal development (Yang et al., 2020). GAP43 is a nerve tissue-specific phosphorylated protein that plays important roles in axonal regeneration, synaptogenesis, and neural function recovery (Fan et al., 2024). Previous studies have shown that GAP43 expression decreases significantly after ischemic stroke (Chen et al., 2020; Gao et al., 2022). In line with previous observations, the GAP43 expression level decreased significantly after MCAO in our study. AAV-sh.ROCK2 treatment promoted axonal growth by increasing GAP-43 expression. High GAP43 expression is associated with axonal extension and synaptic formation (Zhu et al., 2019). SYP is a glycoprotein distributed in presynaptic membrane vesicles and is an important marker of synapsis. Up-regulation of its expression indicates that the synaptic density, synaptic function, and synaptic plasticity are increased, which in turn reflects neuroplasticity. Our results revealed that SYP decreased significantly after MCAO, indicating impaired neuroplasticity. AAV-sh.ROCK2 treatment significantly increased SYP expression, which indicates increases in the number of synapses and neuroplasticity. CRMP2 is a downstream protein of ROCK2, which is expressed mainly in developing neurons (Sun et al., 2020b). It stimulates axonal growth and growth cone elongation by promoting microscopic assembly (Stratton et al., 2020). Overexpression of ROCK2 can lead to phosphorylation of CRMP2, thus inhibiting its binding to microtubules, inducing growth cone collapse, and inhibiting axonal growth. The CRMP2 expression level was significantly increased after optic nerve injury with inhibited ROCK2 expression (Koch et al., 2014), which is consistent with our findings. These results suggest that knocking down ROCK2 expression promotes axonal growth through up-regulation of CRMP2 expression and neural function recovery after stroke. The above results indicate that AAV-sh.ROCK2 treatment promotes synaptogenesis by inhibiting ROCK2 expression.

## Limitations

In this study, we used young male rats to establish an MCAO model. The incidence of ischemic stroke is highest in older adults with diabetes, hypertension, and hyperlipidemia. However, because it is difficult to establish a model in elderly rats, we chose adult male rats to establish a more stable model. Another limitation of this study is that we injected the virus 2 weeks before the MCAO model was established. This type of pretreatment is different from clinical treatment. We can consider injecting the virus after MCAO model establishment to evaluate its efficacy. Intraventricular injection causes secondary injury, and we can evaluate the effect of intracranial transfection by intrathecal injection on small injuries. However, this study confirmed the long-term prognostic effect of ROCK2 inhibition on rats subjected to MCAO, which provides a viable therapeutic method for the treatment of chronic ischemic stroke.

## Conclusion

Compared with traditional ROCK inhibitors, which lack selectivity and have low potency, AAVs have tissue-taxis, transduction specificity, low virulence and immunogenicity, and the ability to promote long-term expression of therapeutic genes *in vivo*, which is why they are common viral gene delivery tools in clinical trials. Our results showed that AAVs carrying the exogenous gene sh.ROCK2 could successfully transfect brain tissue, survive for a long time, and effectively knock down ROCK2 expression. This promotes neurogenesis, synaptogenesis, and neuronal survival after ischemic stroke, which increases neuroplasticity and promotes neural function recovery. Therefore, using AAVs to carry sh.ROCK2 genes that specifically knock down ROCK2 expression in brain tissue may provide a potential method for ROCK2 gene therapy to improve the long-term prognosis of ischemic stroke.

## Data Availability

*No additional data are available*.
